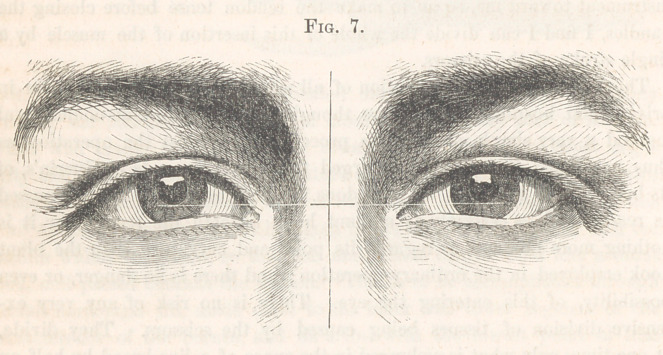# Prominence of the Eyeball, Etc., after the Ordinary Operation for Strabismus; with Description of a New Pair of Scissors for Performing the Sub-conjunctival Operation

**Published:** 1858-03

**Authors:** Addinell Hewson

**Affiliations:** One of the Surgeons of Wills Hospital, Philadelphia


ART. I.—*On the Prominence of the Eyeball, and Sinking of the Caruncle and Semilunar fold following the ordinary operation for Strabismus. With an account of a pair of Scissors for performing the Sub-conjunctival Operation.* By ADDINELL HEWSON, M.D., one of the Surgeons of Wills Hospital, Philadelphia.

EIGHTEEN years have now elapsed since the operation of tenotomy was first successfully performed by Dieffenbach, of Berlin, for the cure of strabismus; and the thousands of cases which were subjected to this mode of cure, immediately on the announcement of his success, by surgeons of undoubted skill, as well as by the hosts of *squint-cutters* who sprung up like mushrooms everywhere, should have early determined, it might naturally be supposed, beyond all doubt, the real value of the operation. Yet this, it would seem, is really far from being the case; for although no one can deny that squint in any direction can be rectified by division of the muscles which give rise to the distortion, and that the operation is recommended by most surgeons, there are many of much experience and sound judgment, at the present day, who declare that, in a large majority of the cases subjected to it, the operation, especially when performed on only one eye, is far from being satisfactory in its results, as it is followed by a peculiar *vacant staring expression* by no means agreeable to a beholder. This effect has alone deterred many afflicted with obliquity from submitting to the operation, and influenced many surgeons in declining to resort to it.

This objection was raised against the operation early in its history, for Chevalier d'Ammon mentions it in a letter to Dieffenbach* as a constant result, even in the most satisfactory cases. And *Calder*, whose *brochure* on the operation was written in 1841, says he considers "this peculiar staring appearance, or seeming largeness of the eye after the operation, as one of its greatest drawbacks."† Then Duffin‡ has well remarked, that "the original obliquity of vision, it is true, may be removed" (by the operation;) "but if in its stead there be substituted a staring, vacant, projecting eye of apparently disproportionate size or a disagreeable leer, or if a mere alteration only in the nature of the obliquity be effected, it is very questionable how far the expression of the eye is bettered by the change."

A reviewer of Mackenzie's essay on the operation, writing in the Medical Gazette, in 1841,§ states, "that in a walk along the streets one may now

* Arch. Général, for 1841, p. 260.

† Calder on Strabismus, London, 1841.

‡ London Medical Gazette for 1839–40, p. 941.

§ Vol. ii. p. 948.

recognize almost as many who have been *cut* as there were two years ago obviously squinting."

This effect of the operation has also been admitted by Lawrence, Mackenzie, Walton, Nelaton, and most who have written anything of late on the subject. Mackenzie, especially, admits that "the eye which has had its adductor divided, presents a greater gap between the cornea and caruncle than natural: the caruncle is more sunk, the lids more open, the eye more prominent and convex at the nasal angle." "If both eyes," he further remarks, "have been operated on, both are rendered more prominent than natural; but being equally so, the circumstance attracts less notice. Where only one eye projects, and the projection is great, the physiognomy is very remarkably and disagreeably affected."* Then Mr. Critchett, who has written very recently on the subject,† is still more positive in the expression of his opinion, and all who are at all familiar with his valuable contributions to ophthalmic surgery, will receive his statements with the greatest confidence as to their accuracy. He says, "the first point that strikes every close observer, even in the most favorable specimens of the operation, where the eye has assumed a perfectly normal position and moves freely, is a certain sinking in and loss of the caruncle, so that the inner part of the globe seems more exposed than that of the opposite eye, and a fossa exists in the place of the caruncle; this, so far," he says, "as my experience goes, is an invariable result of the operation, and explains the circumstance that has often been remarked, that those cases are the most successful in which it has been necessary to operate on both eyes," etc. And for my own part, I must candidly confess, although a warm advocate of the operation, that I have yet to see a case which had been subjected to it—and I have sought diligently for such—in which I could not recognize the fact, often at a glance, and always by close inspection. Finally, such has been the effects of this operation, that a person of no less practical skill and judgment than Mr. Bransby B. Cooper, has "questioned the propriety of operating altogether."‡

It will thus be seen that it is very generally admitted that a disagreeable expression of the eye is a frequent, if not constant sequent of the operation as originally proposed for the cure of strabismus. This peculiar expression is due, as has already been intimated, to increased prominence of the eyeball, and depression or retraction of the semilunar fold and caruncle,—the latter, as Dr. Dix,§ of Boston, long ago observed, giving to the eye "the appearance of a globe lying in the orbit, but detached from it."

* Mackenzie on the Eye, American edition, 1855, p. 381.

† Practical Remarks on Strabismus, published in the London Lancet for 1855, vol. i. p. 479.

‡ Proceedings of the Royal Medical and Chirurgical Society of London, reported in the Lancet for 1840, vol. i. p. 159.

§ American edition of Cooper's Dictionary: Article "Strabismus."

The fact once admitted that such effects follow the operation, it becomes a matter of great importance to determine their cause—or causes, if more than one,—and to determine whether they must necessarily follow the division of the rectus or not, and how they can be avoided.

The opinion early set forth by Herbert Mayo, in regard to the increased prominence, is the one advocated by Mr. Critchett and most other writers. It is, that it is due to the loss of the balance of power between the recti and obliqui muscles—the latter acting, as Mr. Critchett says,* with undue power when one of the recti is divided. If such is the case, the defect is an unavoidable consequence of the operation, and must invariably follow the division of one of the straight muscles, no matter how this division may be accomplished. Is, however, this opinion a mere hypothesis, or is it based on facts? It is said to be based on the *facts*, that the recti, when acting together, have the power to retract, and the two obliqui, by a similar unity of action, to protrude the eyeball. But are these *facts*? In the first place, does the eye, in its normal state, undergo either retraction or protrusion? It certainly does, to a very limited extent. This is shown every time the eyelids are opened or shut—as was pointed out by Sir C. Bell.† Then protrusion, or increased prominence of the ball, is apparent in the staring of amazement. And the retraction has been observed, it is said, during operations on the eye,—as that for cataract. Admitting, then, that such movements can take place in the ball, is it by the action of the straight and oblique muscles that they are produced?

The recti arise, in general terms, from the ligament of Zinn, at the apex of the orbit; and passing forward on either side, are said to embrace a large segment of the eyeball, on which they have their insertion, near the margin of the cornea. If there is nothing to interfere with their action, these muscles must, under such circumstances, have a tendency, whenever they contract, of compressing the eyeball, as well as causing it to retract in the orbit. One of these effects must as necessarily follow as the other, and the contents of the ball must, of a consequence, be disturbed in their relations by every movement given to the eye by these muscles. But it is well-known to every one that such effects do not attend the action of the recti muscles. They are prevented from exerting such influences on the eyeball by the *deep ocular fascia*, first demonstrated fifty years ago by Tenon, and more recently described by Farrell, Hélie, Richet, and others. This fascia, to which Mr. Farrell has given the name of the *tunica vaginalis oculi*,‡ is a fibrous tunic, of a yellowish white color, connected in front with the margin of the orbit, and extending inward and backward to the entrance of the optic nerve into the sclerotic, where it becomes continuous with the dense envelope of that nerve. It

* Op. Cit. † Nervous System of the Human Body, by Sir C. Bell: Edinburgh, 1836, p. 153.

‡ Dublin Journal, vol. xix. Old Series for 1841, p. 337.

forms a covering to the adipose tissue in the bottom of the orbital cavity, and serves as a socket for the eye to roll in. It has six openings, through which the tendons of the muscles escape to reach the sclerotic coat. "These tendons are loosely connected to the edges of these apertures by fine cellular tissue,*" which forms an envelope for them, but in no way interferes with their motions through these openings. Farrell's mode of demonstrating this fascia does not give a correct idea of its relations to the ball, and especially of the position of these openings for the recti muscles. By splitting the lids in a vertical direction, stretching each of the flaps thus formed forcibly away from the eye, and fixing them firmly in these positions, the tissues beneath the conjunctiva are all very much distorted in their relations. By this stretching, the sub-conjunctival layer of the ocular fascia is found drawn away from the ball, when the conjunctiva is dissected off, and appears blended with the deeper layer, and the two seem like one dense membrane extending from the cartilages of the lids to the optic nerve behind the ball; and the openings, or places of exit of the recti muscles, seem at some distance from the ball. A horizontal or vertical section of an eye frozen in the orbit, with all its appendages in their natural position, gives a very different view of the relations of these structures.

FIG. 1.†

Figure 1, drawn by Mr. Daniels with great care and accuracy, from a

* Farrell.

† Figure 1 represents a transverse section of the right eye, with all the reflections of the occular fascia, and its relations to the eyeball, muscles, and lids, faithfully delineated from a dissection made by the author, and still in his possession. The distance between the anterior or sub-conjunctival, and the posterior or deep ocular fascia, is somewhat greater in the drawing than is natural, in consequence of the traction made on these two layers to show the character of the dense cellular tissue which fills up the space between them.

horizontal section prepared in this way, shows the true relations of these orifices for the tendons of the recti. These orifices, if they may be so called, are here seen at the points where the ocular fascia divides into two leaflets, (tracing it from behind forward,) the one to pass outward—speaking of the eyeball as a centre—to the edge of the orbit, the other forward, over the anterior segment of the ball. Of the further reflections and attachments of this fascia we shall have to speak hereafter.

Each one of the recti, just before passing through the orifices in the fascia, divides, as Tenon pointed out, into two tendinous bands. The smaller of these two bands is directed toward the edge of the orbit, and passing behind the tunica vaginalis, or deep ocular fascia, becomes attached to the tissue covering the orbit, by a small flat tendon. The other passes through the tunica, and being directed inward, is inserted into the sclerotica. The smaller portion Tenon calls the *orbital* tendon, the other the *ocular*. Of all the orbital portions, that "of the internal rectus is relatively the largest."*

The influence of these *orbital tendons* can be readily perceived. Placed as they are behind the deep fascia, they are admirably arranged to prevent the recti from compressing the ball and causing it to recede in the orbit. These tendons, aided perhaps by the fascia, have the effect of making the action of the recti on the ball similar to what it would be if these muscles had their origin from the sides of the orbit, while they possess a rapidity and facility of motion greater than could in that case belong to them. These anatomical details certainly do not seem to me to favor the possession of a power by the recti of retracting the eyeball.

But vivisections have been resorted to for the purpose of determining this action of the recti, and Mr. B. B. Cooper's experiment is cited to prove the possession of this retracting power by these muscles. He succeeded in producing permanent retraction of the globe within the orbit in a rabbit, by dividing the two oblique muscles. But is it not verily begging the question, to say that the recti produce this retraction? The experiment proves nothing whatever in regard to the action of the recti muscles.

Then, palsy of these muscles, constituting a form of what is called Luscitas, uncomplicated by any other lesion, also proves that they have nothing to do with the protrusion or retraction of the eye, for in this disease (which is sometimes confounded with strabismus) we have no increased prominence as we should have if the recti, in their normal state, prevented protrusion of the ball.

But does not the deep-seated position of many squinting eyes furnish pathological evidence of the retracting power of the recti? By no means; for *true squint*, it is now well known, is due to a want of co-ordi-

---

* Richet, Traité Pratique d'Anat. Med. Chirurg., p. 307.

nating power, or consentaneous action of the two eyes, rather than to a rigidity or shortening of a rectus of one of them. In all cases of the disease, by closing the straight eye and then desiring the patient to look directly at you with the affected one, he will do so without any difficulty; the eye will move with the utmost promptness in obedience to his will, in every direction. This the patient certainly ought not to be able to do, if the deformity were owing to rigidity or shortening of one of the straight muscles, or paralysis of its antagonist.

Having adduced, as I believe, sufficient evidence that the recti have not the power to effect retraction of the eyeball or prevent its protrusion, we have next to inquire whether the united action of the oblique, when unopposed, can cause its protusion. The anatomical relations of these muscles do undoubtedly favor the idea that they can give rise to such an effect.

The superior oblique, or *obliquus major*, is placed at the upper and inner part of the orbit, and arises about a line from the optic foramen, at the upper and inner part. From thence this long slender muscle proceeds toward the internal angular process, and terminates in a round tendon, which passes through a fibro-cartilaginous ring or pulley, (trochlea,) formed in the edge of the deep ocular fascia, and "attached to a depression on the frontal bone at the inner margin of the orbit." Thus the tendon escapes at this point from behind the ocular fascia. It is then "reflected *outward* and *backward,* and passing between the globe and the superior rectus is inserted into the sclerotica, midway between the superior and external recti muscles, nearly equidistant from the cornea and the entrance of the optic,"* and consequently behind the vertical axis of the eye.

The inferior oblique arises from a minute depression on the orbital process of the superior maxillary bone, just within the inferior margin of the orbit, and close by the external border of the lachrymal groove."† It is surrounded at its origin by the deep fascia, so that it may be said to be entirely in front of that structure. "The muscle then inclines *outward* and *backward* between the inferior rectus and the floor of the orbit, and ends in a tendinous expansion, which passes between the external rectus and the globe to be inserted into the sclerotica at its external and posterior aspect.

As to the action of the muscle, the superior oblique may be regarded as arising from the trochlea, a point vertically opposite the origin of the inferior oblique. With such points of attachment, the combined action of the two must be to draw the ball forward and inward; or if the inward tendency be opposed by the external rectus, they must thrust the ball forward only, or forward and a little outward; but in all instances the movement *forward* will be decided. Their united action must, therefore, be

---

* Sharpey and Quain, p. 318, vol. i., American Edition. † Ibid.

capable of preventing retraction in an opposite direction. This capability was proved by Bransby B. Cooper's experiment, before alluded to, for in that experiment it will be remembered *retraction* occurred on the division of these two muscles.

If it is admitted, then, that the oblique muscles can, by their united action, protrude the ball in a manner more or less forward, and that the recti have no power by united action to move it in an opposite direction, what means, it may be asked, are there of opposing this tendency of the oblique, and causing the eyeball to recede as much as the deep-seated structures of the orbit will allow?

To this question I would answer, that in the eyelids are to be found the chief resistance to this tendency of the oblique muscles, and the power of forcing the eye backward.

These structures, as is well known, consist, beneath the common tegumentary covering and connective tissue, of a *sphincter* muscle, attached most firmly at the inner angle,—two cartilages connected by dense fibrous tissue (part of the ocular fascia) to the orbit, and a reflection of the conjunctiva from the ball of the eye. The two lids differ, however, very much in size, and also in the extent and character of their motion. The upper is much the larger and deeper of the two. Its cartilage is semi-elliptical, and has a depth of six lines at the centre.* This lid descends, in the closing of the eye, below the lower border of the cornea,† and fairly embraces a large portion of the anterior segment of the ball. The inferior lid has a long, narrow, and nearly straight cartilage of about a uniform width of two lines.‡ Its position is horizontal rather than vertical, and it ascends and descends but little in the closing and opening of the eye. The points of juncture of the two lids, constituting the inner and outer angles, are not on the same plane, either vertically or horizontally,—the inner being a little above and in front of the outer. They are both much behind the anterior surface of the eyeball. The whole structure of the lids is more firmly attached at the nose than toward the temple.

The muscle of the lid consists of a broad stratum of elliptical fibres, deriving their origin chiefly from the tendo oculi at the inner angle. "The only points of fixed attachment (to bone) which its fibres possess, are at the inner margin of the orbit; they are free in the rest of their extent, except along the eyebrow, where they are blended with the occipito frontalis and corrugator supercilii.§

The fibres of the orbicularis, curving around the outer angle, have no direct connection with the edge of the orbit at this point; the cartilages, however, have, by a layer of dense fibrous tissue called the external angular ligaments.

---

* Harrison. † Sœmmering. ‡ Harrison. § Sharpey and Quain, p. 335.

All these circumstances seem to me to favor the idea that the lids play an important part in retaining the eyeball in position against the action of the oblique.

Any one, by closely watching the position of the outer commissure of the lids during the acts of shutting and opening the eye, may convince himself that this point (the outer commissure) glides inward, forward, and a little downward, and that the ball recedes somewhat as the eye is closed, and the reverse occurs as the eye is opened.

Then the protrusion of the ball which attends palsy of the orbicularis, as occurred in cases reported by Sir C. Bell,* proves negatively, at least, that the lids prevent protrusion. And a case recorded by Mr. Holthouse† proves the fact in the most positive and conclusive manner. The patient was one "suffering from paralysis of the third pair of nerves. The symptoms were ptosis, external strabismus, dilated pupil, and a total inability to move the eyeball from the position in which it was drawn by the external rectus. "Raising the upper lid gently with my thumb," he says, "I told the patient to close her eye, and in her efforts to do so the orbicularis visibly depressed the globe toward the bottom of the orbit, and at the same time moved the cornea somewhat inward, so that it was made to occupy a situation midway between the external and internal canthi of the eyelids."

And again, Mackenzie, in his work on "Diseases of the Eye," in alluding to a case of luscitas, states a fact singularly conclusive as to the views here advocated. He states that the luscitas had been preceded by exophthalmos and palsy of the side of the face, but that these symptoms had subsided. Here we have the protrusion subsiding with the disappearance of the paralysis of the orbicularis.

These views of the effect of the recti and orbicularis on the protrusion of the ball are not new. They have been urged before, and, among others, by Mr. Holthouse; yet this gentleman, after declaring that the recti have no power to produce retraction, but that this motion of the ball is caused by the orbicularis, appears to have forgotten what he had said, for he subsequently speaks of an antagonism between the obliqui and recti being destroyed, and retraction or protrusion occurring as the consequence of either set of muscles being weakened or strengthened; and remarks that "this has an important bearing on the operation for strabismus, for the division of one of the recti, by weakening the retractive force, relatively increases that of the opposing one; so that some degree of protrusion, under these circumstances, must always be looked for."‡ How well might Mr. Lucas retort on him for this, in his own words; for in speaking, in a subsequent part of his book, of Mr. Lucas's report of the results following the division of the oblique, he says:—"The report of these operations by Mr. Lucas

* Op. Cit., Appendix. † Holthouse's Lectures on Strabismus, London, 1854, p. 8. ‡ Ibid. p. 11.

not only bears out the views advanced in this paper, but is remarkable as showing the little faith which anatomists place in their own accounts of the action of these muscles. Thus, Mr. Lucas having informed us that the action of the inferior oblique is to direct the eye upward and inward, and that of the superior oblique downward and slightly outward, does not hesitate to divide the last-named muscle in order to rectify a convergent squint."

But, to return to the subject, it will be exceedingly difficult for any one, adopting the opinion that the recti have no power to retract the eyeball or prevent its protrusion, to understand why the simple division of the rectus internus should cause increased prominence or protrusion of the ball. Indeed, if the arguments I have adduced in regard to what I am disposed to believe are the chief retaining and protruding powers of the eye, have any truth in them, the simple division of one of the recti must not, necessarily, be followed by such protrusion; and yet I have stated and proved, on the testimony of the most reliable witnesses, that such an effect always follows the operation performed in the ordinary way. Yes; but in the operation are not other parts divided besides the muscle? and may not the division of these be the source of the deformity?

To determine this, let us first inquire what is the character of the wound made in the operations, which have been followed by this defect.

Dieffenbach, in his earliest operations, after making the conjuctiva tense by means of two hooks at the inner canthus, "proceeded to incise the conjunctiva in this part, (the internal canthus,) by the side of the globe, seperated it still deeper from the latter, and then divided the internal rectus thus exposed, with a pair of fine scissors, near to its insertion."*

Of the effect of this mode of operating, I have already given the testimony of Ammon, furnished in his letter to the great Berlin surgeon.

Then Mr. Lucas† (who was the first to publish on the subject in our language) uses a sharp hook to draw the eye out and fix it. This he inserts into the tunica conjunctiva, about two lines, or, two and a half lines distant from the cornea, and on a line corresponding to its transverse axis. He then gently draws forward the conjunctiva, and makes a semicircular incision of this membrane by means of the sharp-pointed scissors, from below upward upon the outer side of the hook, to an extent varying from four to six lines; and, he says, it may sometimes be desirable to make the incision longer.‡ Through this opening he passes a blunt hook from below upward between the tendon of the muscle and the sclerotic, and having thus brought the tendon into view, he divides it with the scissors as close to its insertion as is compatible with the safety of the sclerotic.

---

* Dieffenbach's Letter to Dr. Lomas, Lond. Med. Gaz. for 1839-40, p. 109.

† A Practical Treatise on the Cure of Strabismus or Squint, by Operation. By P. Bennett Lucas, London, 1840.

‡ Ibid. p. 70.

This mode of operating, adopted by Mr. Lucas, although different in its details from that resorted to by Dieffenbach, is essentially the same, it may be said, as regards the direction and character of the incision in the parts covering the muscle, although, it is true, he divides the conjunctiva and subjacent parts nearer to the cornea. Of the effects following this operation we have the testimony, already quoted, of Mr. Duffin, who adopted the method.*

Following close on the publication of Dieffenbach's and Lucas's modes of operating, we find a host of different methods proposed by various individuals. Indeed, it is quite curious, as well as amusing, to see the warfare which was carried on in the journals, in the early history of the operation, as to who was the first to resort to one mode of operating or another; as to the relative value of hooks and elevators, scissors and knives, in the operation; as to who was the first to propose the use of the one or the other; as to who did the operation first in this place or that, etc. Still, amid all these trifling discussions, many useful hints reached the light, and the details of the operation were varied in many ways. With perhaps the exception of the methods proposed by Guerin, Amussat, and Boyer, they are all alike as regards the character and direction of the incision made to expose the tendon of the muscle, and the remarks made in reference to Dieffenbach's and Lucas's may be applied to them all. They all recommend a free division of the parts in a vertical direction, not only for the purpose, in all instances, of exposing the muscle, but often of freely dividing every band of tissue which it was supposed might be binding the eye down in its distorted position; and even of dividing the oblique—one or both—to rectify the deformity.

It must also be admitted that even the methods most in vogue at the present day resemble each other in the vertical direction of the incision. It is true that most surgeons now avoid any very extensive dissection of the globe, or unnecessary disturbance of the ocular fascia, being satisfied with the division of the superjacent tissues, near the insertion of the tendon. Yet, even when all this has been done, it is admitted that "protrusion of the globe, in some degree, occurs in nearly every instance."†

Having detailed just so much of the operation as is sufficient to show the character and extent of the division of the superjacent tissues resorted to, I have now to show how such a division of these parts can give rise to increased prominence of the eyeball and sinking of the semilunar fold and caruncle. But, as I have taken the ground that the tendency to protrusion in the normal state of the eye is opposed by the orbicularis, I have really now to prove that the division of the superjacent tissues of the ball disturbs

* Lond. Med. Gaz. for 1840-41, p. 51.

† Walton, p. 269, Amer. Edit.

the action or weakens the power of the orbicularis, and thus causes the protrusion of the eye; for I have denied that the division of the rectus has anything to do with this protrusion.

This, I believe, can be done by a careful consideration of the *close connection* of the superjacent tissues of the rectus with the *orbicularis*, and especially the connection of the ocular fascia with the latter muscle.

For this purpose I will consider the ocular fascia more in detail than I have yet done. I shall, of course, to do this, be compelled to go over much of the ground I have already been over, and although I may even trace it in the same direction as I have done, the repetition, I hope, will not prove irksome in connection with the new importance attached to its relations.

Tracing this ocular fascia, as Tenon* has done, from the optic nerve forward, we find it forming a complete cup for the ball, and sending off on each side a kind of *ligamentous wing*, as Tenon calls it, to attach the globe to the orbit. "These ligamentous wings are formed by the juxta-position (*l'adossement*) of the portions of this tunic, which pass, the one from the front and the other from the back of the globe."† The anterior leaflet of these "wings" corresponds to what is usually spoken of as the *sub-conjunctival fascia*, the posterior to the *deep-seated ocular fascia*. (See Fig. 1, p. 269.) These two (the sub-conjunctival and the deep-seated ocular fascia) are, however, but separate layers of a continuous structure; for the fascia, traced from the optic nerve, is found, when it reaches where the ocular tendon pierces it, to become much thicker, and divided into two leaflets,—the posterior, and much the stronger, passing off in front of the *orbital* tendons to the margin of the orbit, acquires a firm attachment to this border, and becomes continuous with the posterior margin of the tarsal cartilages for their whole length, and thus forms the ligaments (suspensary ligaments as they are called) of the lids. The other leaflet of the ocular fascia continues forward, and forms a covering to the anterior segment of the sclerotic as well as to the tendons of the straight muscles; and when this leaflet reaches to within about one-twelfth of an inch of the circumference of the cornea it is doubled on itself, and being reflected under the conjunctiva, it forms the sub-conjunctival fascia. (Fig. 2.) Some anatomists have described this layer of fascia as extending close up to the cornea, but I have never been able to detect it nearer than I have described, and I have the authority of Harrison and Jamain that it does not extend close to the cornea. Tracing this layer (the sub-conjunctival) from the ball to the inner angle, we find it passing somewhat obliquely backward, immediately under the conjuntiva, to meet the deeper layer. The two layers, although intimately connected by dense cellular tissue which fills up the space be-

* Recherches sur Anat., Paris, 1806. † Op. Cit. p. 201.

tween them near the ball, become closely blended together in the neighborhood of the semilunar fold and caruncle, to which they have a firm attachment, as can be proved by traction on them. (See Fig 1, p. 269.) Then their united structures become blended with the layer of tissue covering the tensor tarsi, and with "that strong and very tense aponeurosis derived from

FIG. 2.*

the margins of the tendo oculi which extends over the lachrymal sac,"† and which is described by most anatomists as the posterior tendon of the orbicularis, from the firm support it affords the muscle through its true tendon. From this point we find the fascia connected with the tendon, and diffusing itself beneath the conjunctiva of both lids. This is all shown in Fig. 1, p. 269.

---

* An anterior view of the sub-conjunctival portion of the ocular fascia, showing its relations to various structures of the eye. The conjunctiva has all been removed except a small portion around the edge of the cornea, which, however, has been carefully dissected up to that edge. The fascia is left in situ on the outer segment of the ball, showing how close it extends to the edge of the cornea, and its manner of reflection from the ball to the margins of the orbit to be diffused over the surface of the lids beneath the conjunctiva. On the upper, lower, and inner segments it has been partly detached from the globe. The portion from the inner segment has been put upon the stretch in a vertical direction, and the ball located so as to bring into view the tendon of the internal rectus at its insertion, and to show the portion of the deeper layer interposed between the tendon and the sclerotic, forming the posterior border of the opening in this deep layer for the transmission of the ocular tendon of the muscle. The connection of the sub-conjunctival layer with the caruncle can readily be seen in this picture. An incision like that in the ordinary operation has been made through both layers of the fascia, to expose the belly of the muscle.

† Harrison.

On the outside, as well as above and below, these two layers of fascia pass in the same way from the ball, to meet and become blended near the edge of the orbit, and thence connect with the lids. In the lids, at the temporal angle, they form the external angular ligaments of Winslow.

It may be thus seen that this fascia is blended with more than one structure at the angles of the orbit; but that it is especially connected with the semilunar fold and caruncle any one can satisfy himself by making a dissection of the eye from behind, and removing all the adipose tissue and other intervening structures until nothing is left but the fascia, and then drawing it backward, he will readily perceive that he causes these parts to recede. Then, again, by dividing this fascia, with the conjunctiva, in a vertical direction, as in the ordinary operation for strabismus, I have found that I could by traction on both lids draw the outer end of the tendon some little forward, much more so than I was able to do before dividing these parts. I also found that my ability to draw this point forward was in direct ratio with the extent of the vertical division of the superjacent parts and the nearness of the incision to the caruncle.

Finally, a careful examination of an eye which has been operated on for strabismus in the ordinary way, will show that this very change has taken place in the position of the tendon. This may be seen in Fig. 3, copied from a photograph taken recently of a patient operated on by me some four

FIG. 3.

years since. Only one eye, the right, was operated on, and the contrast in the position of the tendons of the two orbicularies is very remarkable. It might be supposed that this appearance of the position of this tendon of the eye operated on was due to the increased depth of the fossa at the inner angle; that the change of the position of the tendon was more apparent than real. Viewing the two tendons, however, from a position in which the fossa cannot have any effect on the impression produced of the situation of the tendons, or when the eyes are both closed, it will be recognized that there is a real difference; that the outer end of the tendon of the right eye does really jut forward a little. But, admitting this change in the position of the tendon, is it not, it may be urged, due to the protrusion of the eyeball, which, putting the orbicularis on the stretch, drags the end of the tendon forward? Is the tendon, however, capable of being moved in this way by the orbicularis? If it was it should come forward every time the muscle is forcibly contracted; but that such does not occur any one can satisfy himself by placing his finger firmly on the tendon, and then forcibly closing the eye. He will then perceive that the tendon remains fixed in its original position, although the fibres of the muscle may be so puckered up as to overlap it. I have not been able to discover that this effect of the operation on the position of this tendon has ever been pointed out before; but since my attention has been called to it, during the past year, I have noticed it as an invariable result; and whenever I have attracted the attention of others to it they have expressed surprise that they had not observed it before, it was so patent.

This disturbance of the *tendo oculi*—the only fixed point of attachment of the *sphincter* muscle of the lids—must have an important influence on the action of that muscle; it must weaken its power to prevent protrusion of the ball, for the nearer this insertion of the muscle is brought to the plane of the anterior surface of the globe of the eye, the less must be its ability to press that globe back. The sinking of the caruncle may be in part only apparent from this position of the tendon. The internal angle of the lids coming forward might give the effect of this little body being more depressed than it really is. Still I am of opinion, from careful observation, that its position is somewhat affected by the operation. It seems to me to be flattened or stretched out, and by pulling on the inner end of either the upper or lower lid of an eye which has been operated on for squint, I have noticed that I could move this body more sensibly than in the natural eye. Of the real change of the position of the semilunar fold there can be no doubt. Both the caruncle and fold, I am satisfied, are disturbed by the division of the conjunctiva and ocular fascia. It was supposed that the disturbance of these parts (the semilunar fold and caruncle) were due only to the nearness of the incision to them. The incision was, therefore, made close to the cornea, and although much more satisfactory results were obtained by this, still the evils of the operation were not entirely prevented, they were only lessened. It is a well-known fact that Mr. Haynes Walton* has advocated operating in this way by a very small vertical incision directly over the insertion of the tendon of the rectus, and just extensive enough to reach and draw out the tendon with the blunt hook. This, how-

* Op. Cit. p. 260.

ever, he does not seem to have found sufficient, for more recently we learn of his closely stitching up the wound after the division of the muscle. Then Hélie,* Amussat, and Lucien Boyer† resorted to a horizontal cut in the conjunctiva and fascia, along the border of the muscle, to prevent the effects of the operation on the eyeball, semilunar fold, and caruncle; and for the same purpose Jules Guerin proposed his sub-conjunctival method.‡ This sub-conjunctival division of the tendon of the rectus has been strongly advocated by Mr. Brooke in a paper read before the Royal Medical and Chirurgical Society of London,§ and recently by Mr. Norman in a communication to the North London Medical Society, at its meeting, Dec. 14, 1856;|| and it has also met with warm support during some years from Mr. Critchett and his colleagues, at the Royal London Ophthalmic Hospital, Moorfields.¶

From such testimony there can be but little, if any, hesitation in believing that the evil effects of the ordinary procedure of Dieffenbach and others are greatly diminished by the sub-conjunctival division of the muscle. And from the same sources for my evidence, as well as from personal observations, I am led to the conviction that the diminution of these effects is in an inverse ratio with the extent to which the fascia is disturbed.

Thus, *en resumé*, I have, at least, attempted to prove the following propositions in reference to the prominence of the eyeball—sinking of the semilunar fold and caruncle—following the ordinary operation for strabismus.

1. That these effects are not due to a disturbance of the balance of power between the recti and obliqui muscles, for the simple reason that no such balance of power exists between those muscles.
2. That the recti have no power to retract or prevent protrusion of the eyeball.
3. That the obliqui can protrude the ball.
4. That the action of the obliqui is opposed by the orbicularis.
5. That the power of the orbicularis to oppose the obliqui is weakened by the extensive vertical division of the conjunctiva and ocular fascia, which, especially the latter, play an important part in sustaining it in its action.
6. That in the ordinary operation these structures are so disturbed as to cause the evils spoken of.

As corollaries to the foregoing propositions I would urge—

*a.* That these evils are not an unavoidable consequence of the division of the rectus.

---

* These sur l'Operation du Strabisme. Paris, 1841.

† Récherches sur l'Operation du Strabisme, 1842–44, 1 vol. 8vo., avec 12 planches.

‡ Memoir sur la Myotomie Oculaire, par la Méthode sous Conjunctival; Gazette Medicale, 1842.

§ Proceedings reported in the Lancet for 1846, vol. i. p. 159.

|| Lancet for 1857, vol. i. p. 67.

¶ London Lancet for 1855, vol. i. p. 479.

*b.* That they can be prevented by a sub-conjunctival division of the muscle.

It must, of course, be for others to determine whether I have been successful in sustaining the truthfulness of these propositions or not; but their admission or denial will not necessarily affect the character of the statements contained in the corollaries, for these statements may be in accordance with the truth, though they are not legitimately deduced or founded upon true premises. They may be sustained by observation and experience, the great touchstones, after all, in our science; for no matter how specious our arguments may be, or how plausible our deductions, if they are not sustained by these they are worth nothing. I have some such testimony to bring forward in support of these statements; but before offering it I will describe the means by which I have divided the tendon of the rectus without disturbing the fascia, and my manner of performing the operation.

For the sub-conjunctival operation I use a pair of scissors, which I devised some time since, and which were made by Mr. Kolbe, from a model I had furnished him. They are represented of full size in Fig. 5, and are

FIG. 4. FIG. 5.

peculiar in their appearance. They consist of a male and female blade, connected together by a compound sliding joint. These blades have a curve of a circle of three-fourths of an inch radius, and are sharpened on opposite sides for the distance of a little over half an inch from their points. The point of the male blade is blunt—that of the female quite sharp. The latter blade slides over the former on two flat-headed screws, which fit accurately in the grooves of this blade, and are capable of being tightened so as to prevent all irregularity of motion. The rings on the handles are arranged for convenience of using the instrument. When the handles are widely separated, as in Fig. 6, the point of the female blade is buried on the side of the male, and as they are closed this point of the female blade comes up in front of the cutting edge of the male, until there is a space of a line in width between the point and this edge, (see Fig. 4.) This point then ascends, preserving always the space of a line between its edge and the cutting edge of the male blade, until the two points are on a level. Then the female blade slides toward the male, and their edges cross, and the whole of the female blade is buried on the side of the

FIG. 6.

male. The whole of this complicated movement is effected by simply pressing the handles together as in closing an ordinary pair of scissors. When well made, the scissors will always close with the utmost regularity and facility; but from the nature of the motion between their two blades, they cannot be readily opened while held by one hand, nor is this at all necessary, for they are to be taken in the hand ready for use, with the handles widely separated, so that the male blade can be used like a blunt hook, and passed beneath the tendon. This is to be accomplished through a small horizontal slit, made close to the inner and lower segment of the circumference of the cornea, and on a line with the lowest point of that structure. A cut of a quarter of an inch in the conjunctiva, in this position, has enabled me to pass the point of the scissors directly between the sclerotica and its cup-like envelope of the ocular fascia, and reach the tendon of the internal rectus without much disturbing the fascia. It will be remembered that this ocular envelope does not, according to Harrison and Jamain, extend close up to the circumference of the cornea, before it is reflected under the conjunctiva, to form the sub-conjunctival fascia; and my own experience leads me to the belief, as before stated, that it does not approach within a line, if it always reaches so near to the cornea. My purpose, therefore, in making the slit in the conjunctiva, as described above, is to get between the edge of the fascia and the cornea. It does not materially affect the results of the operation if this horizontal cut should involve the edge of the fascia, as I have more than once made it do; but it is desirable, I think, to avoid any unnecessary cutting in the operation.

To make this incision I pinch up the conjunctiva, at the point indicated, with a toothed forceps, and divide it with a pair of sharp-pointed scissors. The opening made, I still retain my hold of the conjunctiva with the forceps, and introduce the male blade of the squint scissors through the slit, and insinuating this carefully between the sclerotica and the fascia, I pass its point back to a depth of nearly half an inch; then sweeping it up, and making it hug close to the ball, I slide the blade without difficulty beneath the muscle, and bring its point above the upper border. This done, and there is no difficulty in effecting it, I complete the operation by simply bringing the handles of the instrument together, taking care, as I do so, that the point of the female blade does not catch in the edge of the wound in the conjunctiva. Of this I find there is no danger, if the instrument is laid flat on the sclerotic. The two blades completely divide all the tendon embraced between them, and if I have taken care to carry the point of the male blade above the upper border of the tendon, and to draw the instrument toward me, so as to make the tendon tense before closing the handles, I find I can divide the whole of this insertion of the muscle by a single stroke of the scissors.

These scissors, in the estimation of all who have seen me use them in private and hospital practice, are thought to render the sub-conjunctival method a very simple and certain procedure. Against the operation, as thus performed, there cannot be urged the objections made to Guerin's, of its being either uncertain or hazardous.* The male blade can be passed as readily and as certainly as a blunt hook beneath the tendon, for it is nothing more nor less, as regards its point and curvature, than the blunt hook employed in the ordinary operation; and there is no danger, or even possibility, of this entering the eye. There is no risk of any very extensive division of tissues being caused by the scissors. They divide, at one time, only what is embraced in the space of a line broad by half an inch long; and if they are properly used—that is, laid flat to the sclerotica—and care is taken to get the point of the female blade beneath the fascia, they will divide nothing but the tendon. Of this I have again and again satisfied myself by experiment on the dead subject.

Then, again, with these scissors there is not the same difficulty or uncertainty in dividing the insertion of the muscle as there is in the plan proposed by Mr. Critchett, and which is already known to the readers of this journal, as the whole of his paper published in the Lancet, in 1855, was recently stolen by one of his countrymen, and published as an original article, in the North American Medico-Chirurgical Review for March, 1857.

* Liston's Lectures, American Edition, 1846, p. 107.

Mr. Critchett's mode of operating, it will be remembered, consists in passing a blunt hook beneath the tendon through a small horizontal cut in the conjunctiva and fascia, at the lower border of the muscle. "The blades of the scissors must then be passed in through the opening, and, by a succession of small cuts, the tendon may be readily divided between the hook and the insertion into the sclerotic, and close to the latter." He remarks, however, that "some little difficulty in making a complete division is experienced, when the insertion of the tendon is rather broad, in reaching its upper edge; and, when that is the case, I make a small counter-opening," he says, "in the conjunctiva, corresponding to the upper border of the muscle, I introduce the hook from above, and, having passed it beneath the remaining slip of tendon, divide it with the scissors in the same direction."* The length of the blades, and their curve, in my scissors, are sufficient to avoid the necessity of resorting to any such expedient as that described above by Mr. Critchett, to completely divide the tendon when it has a broad insertion. And in this respect, at least, the operation with them possesses an advantage over that proposed by Mr. Critchett.

FIG. 7.

The success attending the use of these scissors has thus far been in strict confirmation of the views I have advanced as to the defects following the ordinary operation. It is now a year since they were devised, and I have in that time had the opportunities of employing them nine times; and in none of the cases in which I have employed them have I been able to detect any sinking of the semilunar fold or caruncle, or any increased fullness of the eye—and for these defects I have made diligent search. I have carefully noted the amount of separation of the lids, and the relations of the semilunar fold and caruncle, before and after the operations; so that, as far as they go, these cases furnish reliable testimony of the results. But

* Lancet for 1855, vol. i. p. 507.

I have as yet had only a single case—such cases are rare—in which the division of one of the internal recti was found sufficient to relieve the squint. This was an outdoor patient of the Wills Hospital; and as no memoranda are taken at that institution of the addresses of such patients, I am not now able to obtain a photograph of her; and, in lieu of her picture, which would furnish such a very positive evidence of the success of the operation, I offer a picture of one of the other cases, (Fig. 7,) as from it, at least, some idea may be formed of the success I have met with. In this case, as well as in the others, the amount of separation of the eyelids, and the position of the semilunar fold and caruncle, were carefully noted before and after the operation, as the only positive means of determining, when both eyes are subjected to it, that no change has been produced in these respects by it. In all these respects the eye is the same as it was before the operation.

Those who are in the habit of following the advice of Lucas, Duffin, and others, to freely divide the fascia in the event of the division of the internal rectus not proving sufficient to immediately remove the deformity, may urge the necessity of dividing that structure as an argument against the method here recommended. But if the existence or persistence of squint is due in any degree to the contraction or binding down of the fascia, how could a patient straighten the eye and move it at will in all directions, as he can do on closing the apparently sound eye. The truth is that, in a very great majority of cases, the disease is one mutually affecting the internal recti of both eyes, although it *appears* to be confined to one only; and Mr. Elliott, of Carlisle, England,* long since laid it down as a rule to divide the second adductor in cases where "parallelism is not perfectly restored by division of the first." This rule has been endorsed by Mackenzie and others, who do not advocate or (as far as my knowledge goes) perform the sub-conjunctival operation; and I cannot better conclude this paper, which has already extended far beyond the limits it was originally intended to possess, than by quoting the remarks of the eminent physiologist, Dr. Carpenter, on this point. He says, "that he is well convinced, from repeated observations, that those surgeons are in the right who have maintained that, in a large majority of cases, strabismus is caused by an affection of *both* sets of muscles or nerves, and not of one only; and that it then requires, for its perfect cure, the division of the corresponding muscles on both sides. Cases will be frequently met with in which this is evident—the two eyes being employed to nearly the same extent, and the patient giving to both a slight inward direction, when desired to look straight forward. In general, however, one eye usually looks straight forward, while the other is greatly inverted; and the sight of the inverted eye is frequently affected to a considerable degree by disuse; so that, when the patient voluntarily

* Edinburgh Medical and Surgical Journal, vol. lv. for 1841, p. 370.

rotates it into its proper axis, his vision with it is far from being distinct. Some surgeons," he further remarks, "have maintained that the inverted eye is usually the only one at fault, and consider that the division of the tendon of its internal rectus is sufficient for the cure. They would even divide its other tendons, if the parallelism be not restored, rather than touch the other eye. The author is himself satisfied, however, that the restriction of the abnormal state of the single eye is the exception, and not the rule, in all but very slight cases of strabismus; and to this opinion he is led both by the consideration of the mode in which strabismus first takes place, and by the results of the operation which have come under his notice. If the eyes of an infant affected with cerebral disease be watched, there will frequently be observed in them very irregular movements—the axes of the two being sometimes extremely convergent, and then very divergent. This irregularity is rarely or never seen to be confined to one eye. Now, in a large proportion of cases of strabismus, the malady is a consequence of some cerebral affection during infancy or childhood, which we can scarcely suppose to have affected one eye only. Again, in other instances, we find the strabismus to have resulted from the constant direction of the eyes to very near objects, as in short-sighted persons; and here, too, the cause manifestly affects both. Now it is easy to understand why one of the patient's eyes should *appear* to be in its natural position, while the other is greatly inverted. The cause of strabismus usually affects the two eyes somewhat unequally, so that one is much more inverted than the other. We will call the least inverted eye A, and the other B. In the ordinary acts of vision, the patient will make most use of the least inverted eye, A, because he can most readily look straight forward or outward with it; but, to bring it into the axis, or to rotate it outward, necessitates a still more decided inversion of B. This remains the position of things: the patient usually looking straight forward with A, which is the eye constantly employed for the purposes of vision, and frequently almost burying under the inner canthus the other eye, B, the vision in which is of very little use to him. When, therefore, the tendon of the internal rectus of B is divided, the relative position of the two is not entirely rectified. Sometimes it appears to be so for a time; but the strabismus then begins to return, and it can only be checked by division of the tendon of the other eye, A; after which the cure is generally complete and permanent."*

NOTE.—Since the greater part of this article was written, I have learned from my friend, Dr. Emil Fischer, that Professor Graëfe, of Berlin, has expressed the same or similar views in regard to the action of the orbicularis and recti, in a recent article published by him in his Arch. of Ophthalmology, for October,

* American edition of Carpenter's Principles of Human Physiology.

1857. Of this fact I cannot certify of my own knowledge, as I possess no acquaintance whatever of the German language. But, if such is the case, it will be no little gratification to me that the views I have attempted to develop should be advocated by so very eminent an authority as Professor Graëfe.

## Figures and Tables

**Fig. 1. f1:**
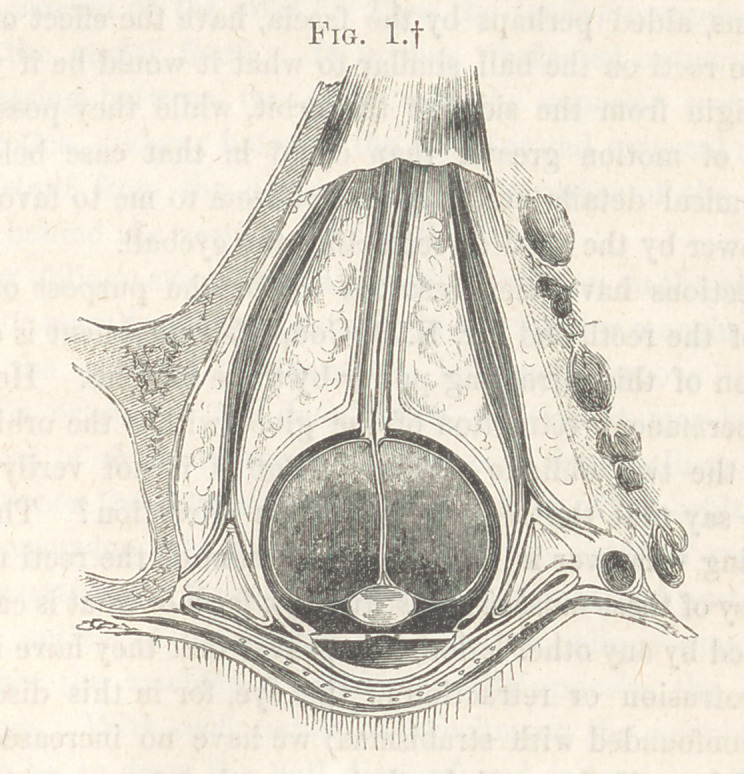


**Fig. 2. f2:**
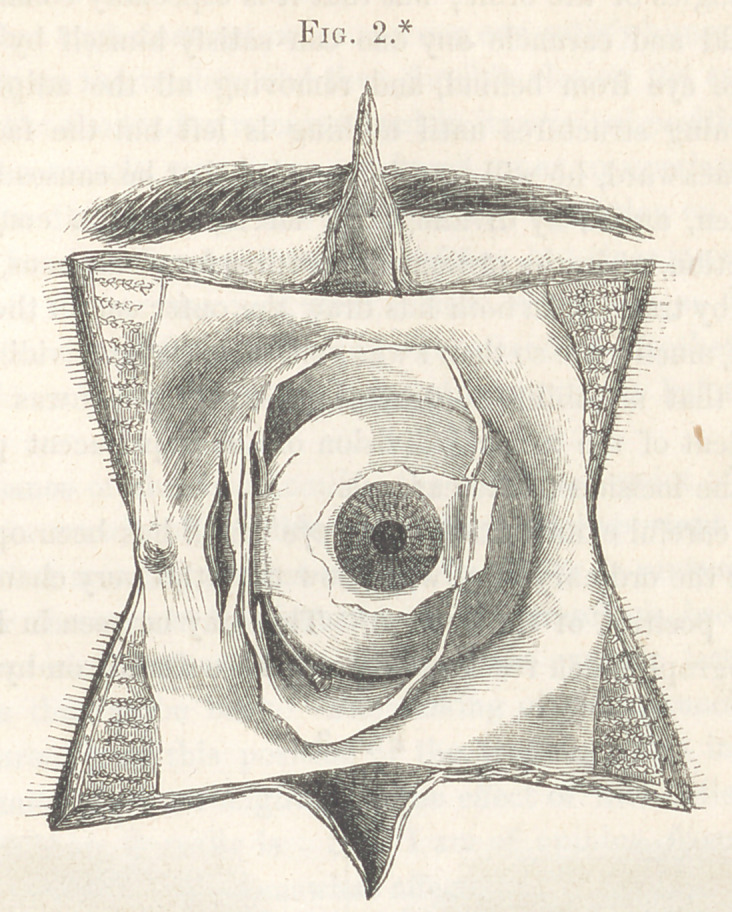


**Fig. 3. f3:**
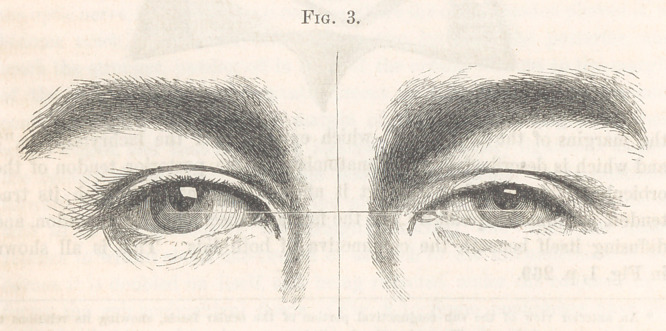


**Fig. 4. Fig. 5. f4:**
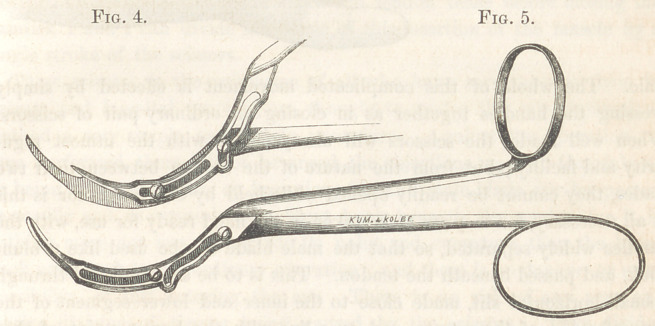


**Fig. 6. f5:**
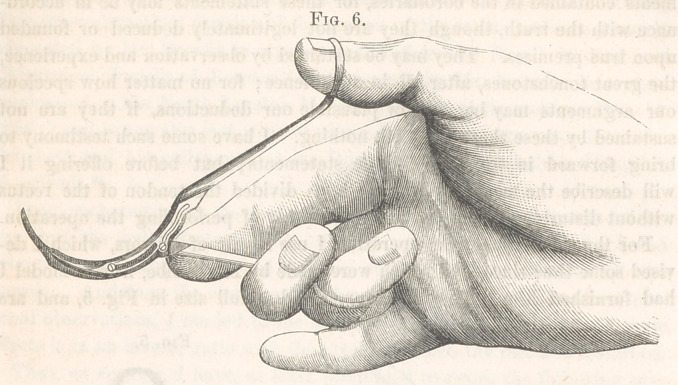


**Fig. 7. f6:**